# Assessing cardiac function in the single ventricle circulation: Kinetic energy ejection fraction

**DOI:** 10.1186/1532-429X-17-S1-O57

**Published:** 2015-02-03

**Authors:** James Wong, Radomir Chabiniok, Kuberan Pushparajah, Eva Sammut, Shane M Tibby, David Celermajer, David Nordsletten, Daniel Giese, Tarique Hussain, Gerald F Greil, Tobias Schaeffter, Reza Razavi

**Affiliations:** 1 KCL, London, UK

## Background

Those with single ventricle circulations often have a normal ventricular volumetric ejection fraction (VV EF) which is not in keeping with their symptoms and prognosis. Recently four-dimensional flow (4Dflow) magnetic resonance imaging (MRI) has been used to extract parameters of intra-cardiac kinetic energy (KE). There has been recent interest in measuring ventricular KE in health and disease to see if this could provide an additional tool in the assessment of ventricular function. We propose a new simple measure of ventricular function based on kinetic energy, the kinetic energy ejection fraction (KE EF), and assess its usefulness in patients with single ventricle circulation.

## Methods

Kinetic energy measured by 4D flow MRI was prospectively quantified in 41 patients with a single ventricle circulation (aged 0.5 - 28 years) and compared to 43 healthy volunteers (aged 1.5 - 62 years) who acted as negative controls, and 14 patients with left ventricular (LV) dysfunction (aged 28 - 79 years) who acted as positive controls. The ratio of ejected kinetic energy to total kinetic energy was used to devise KE EF.

## Results

**Figure 1:**
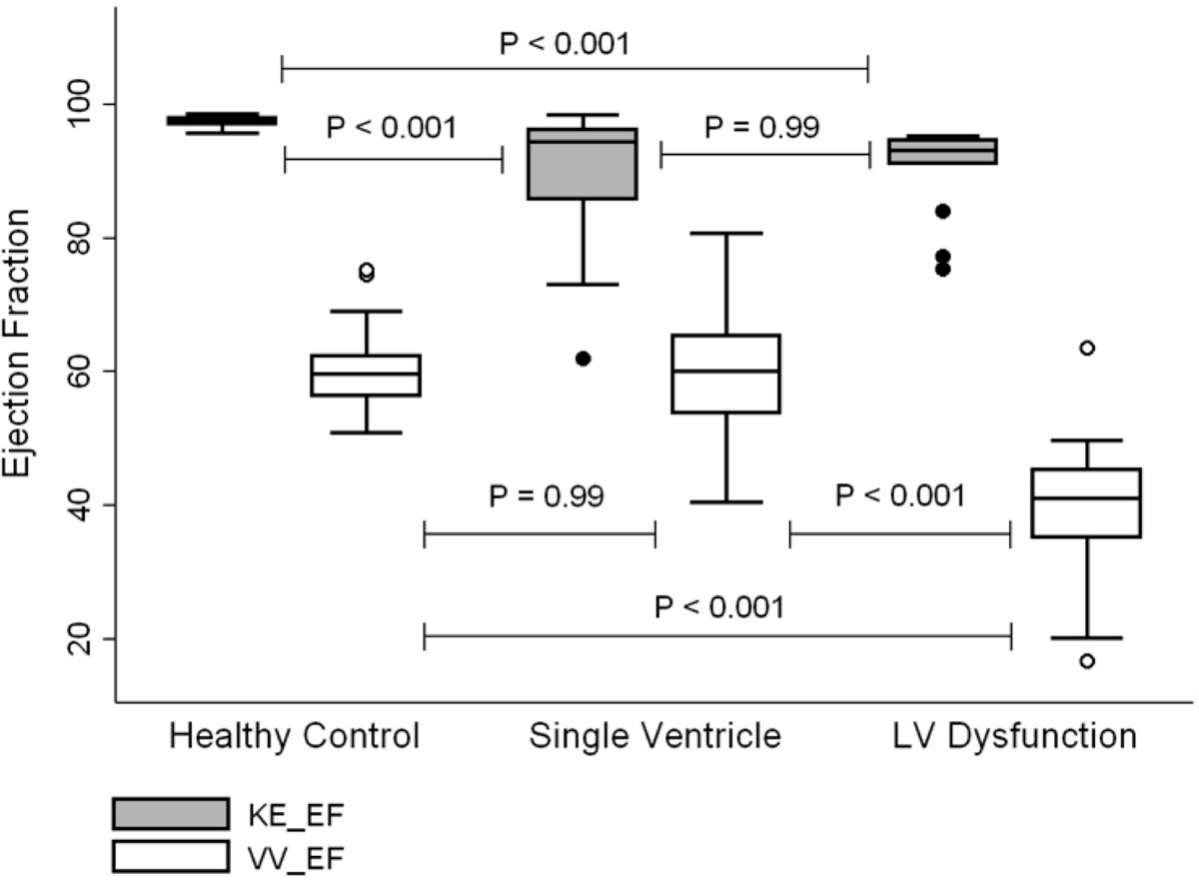
Box and whisker plots showing relationship between patient group and ejection fraction (kinetic and volumetric) for all patients. P values are shown for inter-group comparisons, and are calculated post hoc from the Generalized Linear Model

Figure 1 shows the MRI derived volumetric and kinetic energy parameters. The KE EF has a smaller range than VV EF in healthy subjects (97.3 +/- 0.8% vs. 60.1 +/- 5.5%). LV dysfunction causes a fall in KE EF (90.0 +/- 6.7; *p* = 0.0001). Although VV EF in healthy LVs and single ventricle hearts did not differ (*p* = 0.99) the respective KE EF was significantly lower (91.0 +/- 8.0%; *p* < 0.0001) with a wider range. Those reporting the greatest symptomatic impairment (NYHA II) had lower KE EF than asymptomatic (NYHA I) subjects (*p*= 0.0067) whilst in comparison VV EF remained unchanged (*p* = 0.54).

## Conclusions

Kinetic energy parameters offer new insight into the function of the heart. KE EF is a marker of healthy cardiac function displaying a very small range of values in comparison to the broader range shown in VV EF. In those with single ventricle circulations it identifies a spectrum of diminished cardiac performance corresponding to a patient's perceived exercise tolerance. This is particularly useful in conditions such as single ventricle physiology where standard ejection fraction values are often normal. Further work to determine its relationship with other prognostic outcomes would allow full implementation as a clinical tool.

## Funding

N/A.

